# A missing link: The interdependence between sugar-sweetened beverage and cigarette consumption from China

**DOI:** 10.1371/journal.pone.0316891

**Published:** 2025-01-24

**Authors:** Lu Liu, Kevin Chen

**Affiliations:** 1 Institute of Agricultural Economics and Development, Chinese Academy of Agricultural Sciences, Beijing, China; 2 China Academy for Rural Development, Zhejiang University, Hangzhou, Zhejiang, China; 3 International Food Policy Research Institute, East and Central Asia Office, Beijing, China; University of Modena and Reggio Emilia: Universita degli Studi di Modena e Reggio Emilia, ITALY

## Abstract

Sugar-sweetened beverages (SSBs) and cigarettes are addictive substances and addictive substances are often related in consumption with each other. However, the potential interdependence between SSB and cigarette consumption has not been explored in the literature. As SSB and cigarette consumption have posed a great threat to individual health, the knowledge of such interdependence is critical for policymakers to design and coordinate government interventions. We thus employed Heckman sample selection model and simultaneous equation model to identify and validate the interdependence between SSB and cigarette consumption across subgroups exhibiting different smoking behaviors with individual-level data from the China Health and Nutrition Survey (CHNS) during the period from 2004 to 2011. We find that SSBs and cigarettes are complements in that individuals who ever smoked are more likely to consume SSBs frequently with higher level of SSB intake eventually and SSB intake of current smokers increases along with the amount of cigarettes smoked. SSBs and cigarettes are also substitutes in that former smokers are more likely to consume SSBs compared with current smokers. The complementary relation observed among current smokers implies that government interventions targeting one of the two goods may yield a double dividend effect on health whereas the substitutable relation displayed by former smokers suggests that the health effect of interventions designed to reduce the consumption of one good may be tempered by an elevated demand for the other.

## 1 Introduction

Sugar-sweetened beverage (SSB) and cigarette consumption have been widely recognized as major causes of individual health problems with undesired externalities such as imposing heavy burden on social healthcare costs and therefore necessitated government interventions [1, 2]. As the two are traditionally categorized as different consumption goods, government interventions aiming at reducing cigarette and SSB demand are often introduced separately. However, cigarettes are widely recognized as addictive substances [3] and SSBs are also addictive with emerging evidence from physiological [4–6] and economical [7–9] research. The interdependence between the consumption of addictive substances, such as cigarettes, alcohol and marijuana, has long been identified [10–22], which gives rise to the question that whether SSBs and cigarettes are interdependent in consumption? However, this question has not been explored.

Understanding such interdependence is critical because many countries have struggled with the overconsumption of SSBs and cigarettes. As long as SSBs and cigarettes are correlated, the demand equation for one should incorporate the price for the other or will yield biased estimates of price elasticity [12, 16]. Second, the interdependence between the two indicates that the public policy for one is unlikely to be independent of the other [12]. When SSBs and cigarettes are complements, an increase in the price of one may lead to a decline in the consumption of both at the same time. Correspondingly, a decline in the price of one may encourage individuals to consume more of both, which may cause a rise in health risks. Given the complementary relations, policymakers only need to trace the consumption of one good to understand that of both. Furthermore, the health effect of interventions targeting at reducing the demand for one may be underestimated given the reduction in demand for the other. In the alternative scenario where SSBs and Cigarettes are substitutes, an increase in the price of one accompanies an increase in the consumption of the other. Thus, the health effect of interventions aiming at reducing the consumption of one might be mitigated. Especially, as fiscal policies are recommended as effective tools to reduce SSB and cigarette consumption [23, 24], knowledge of the cross effects is valuable to policymakers to better coordinate diet interventions with other policies in terms of fiscal revenue or expenditures and health effect.

**Fig 1:**
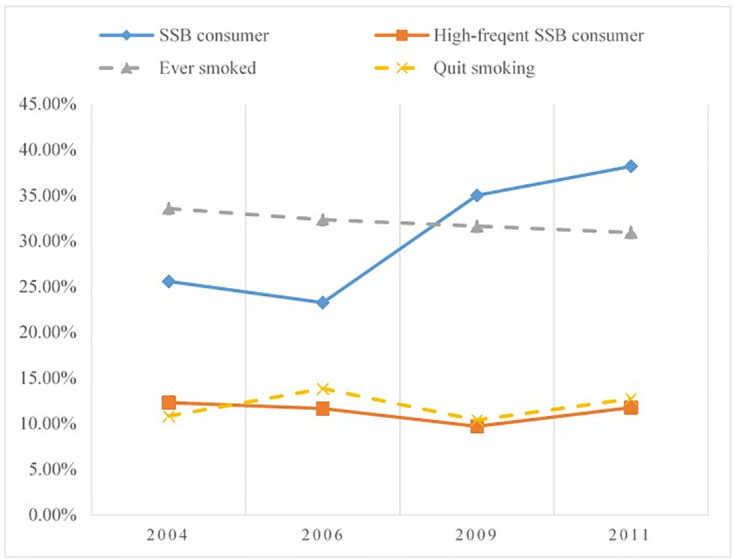
SSB and cigarette consumption during 2004 to 2011.

This study aims to identify how SSBs and cigarettes are related in consumption with the individual-level data from the China Health and Nutrition Survey (CHNS) over the period from 2004 to 2011. With the largest smoking population and a rapid increase in SSB consumption, China is a good example to explore the correlation between the consumption of the two. As for cigarettes, China accounts for 40% of the world’s cigarette consumption [25] and has implemented multiple public policies and legislations to weaken the adverse effects of cigarette consumption on health since 2005 [26]. Consequently, cigarette consumption has gradually declined over the years. While in the same period, SSB consumption has surged, emerging as a significant indicator of dietary imbalance, which are considered a major contributor to the incidence of chronic diseases and fatality in China [27]. This contrasting trend is illustrated by CHNS, as shown in Fig 1 (Section 4.2). One possible explanation behind the rocketed SSB demand may be that SSB consumption in China is rationally addictive [9], that is Chinese consumers are inclined to consume more SSBs than they did in the past to obtain the same level of utility [28] and take in excessive added sugars eventually. Excessive sugar intake is associated with increased incidences of overweight/obesity, dental caries, type 2 diabetes etc. [27, 29] and thus necessitates the government to introduce diet interventions to reduce SSB consumption. Under this circumstance, whether and how SSBs and cigarettes is related in consumption are very important for policymakers to make informed decisions in the coordination of intervention policies.

This study adds to the extant literature by identifying the interdependence between SSB and cigarette consumption with individual-level data from China. To our knowledge, it is the first to explore such a correlation as previous studies mainly examined the addictiveness of cigarettes and SSBs separately [8, 9, 30, 31]. This study also contributes to providing empirical evidence for the speculation from extant studies that addictive substances could be complements or substitutes due to group differences [12, 16, 19]. Our study helps policymakers to better coordinate diet interventions with other public policies and enriches the literature concerning the interdependence between addictive substances in consumption.

The rest of the paper unfolds as follows. Section 2 reviews literature regarding the interdependence between the consumption of cigarettes and alcohol, and the addictiveness of SSBs. Section 3 outlines the empirical strategy. Section 4 describes sample selection, summarizes the data and illustrates econometric models applied in this study. Section 5 presents the results and analysis. Section 6 concludes and discusses policy implications.

## 2 Literature

### 2.1 The interdependence of cigarettes and alcohol in consumption

A body of literature has empirically examined the interdependence of addictive substances in consumption, especially for cigarettes and alcohol. The cross-price elasticities are significantly negative between cigarettes and alcohol in consumption, implying complementarity between smoking and drinking [10, 17, 19]. The complementary relation between the amount of cigarettes smoked and alcohol intake daily was also captured by Tauchmann et al. (2012) and Ren et al. (2020) for German and Chinese separately without price information. Furthermore, Moore (1996) found that the increase in beer tax could significantly reduce respiratory cancer mortality, identifying the complementary effects of smoking and drinking from the perspective of health. However, cigarettes and alcohol have also been found to be substitutes. The cross-price elasticities estimated by Goel & Morey (1995) and Decker & Schwartz (2000) were positive, implying substitutability between smoking and drinking.

The paradoxical conclusions regarding the correlation between cigarettes and alcohol consumption may be rooted in the heterogeneity across cultures and countries [21] while group difference is another plausible explanation. Decker & Schwartz (2000) found that although an increase in beer price would increase cigarette consumption for those who continue to smoke, reflecting substitutability, but may also encourage individuals who smoke occasionally to quit smoking, reflecting complementarity. Moreover, the magnitudes and directions of the cross effects for cigarette and alcohol are found to be asymmetry, which may originate from the asymmetry in the numbers of individuals who only smoke or drink and those who smoke and drink at the same time [12, 16, 19], i.e. the group difference.

Except for cigarettes and alcohol, the substitutability between alcohol and marijuana has also been identified with empirical evidence [11, 15]. Why does the demand for different addictive substances interact? Different theories from medical research are proposed to explain the mechanisms behind such interdependence [16, 32]. First, the correlation may result from individuals’ addictive personality because cigarettes and alcohol are often consumed by the same person, indicating complementarity. An alternative explanation is the oral drive hypothesis which postulates that individuals achieve a balance between smoking and drinking and smoking less will lead to drinking more or vice versa, suggesting substitutability. Another explanation is the learning-based hypothesis which holds that doing one will activate a drive to do the other and thus indicates complementary relations between smoking and drinking. Finally, the pharmacological interaction between cigarettes and alcohol was also proposed as a potential contributor. Although the drive behind the correlation between addictive substances in consumption remains far from clear, extensive empirical evidence has emerged to support such correlation.

### 2.2 The addictiveness of SSBs

SSBs, high in added sugar, are addictive substances based on evidence from physiological research [4, 5]. Human beings generally have an embedded desire for calorie-dense foods (i.e. rich in refined carbohydrates such as added sugar), which stems from the long fight with famine through the evolvement, and therefore tend to develop dependence syndromes similar to tobacco or alcohol in the consumption of such foods [6]. In light of the description of dependence syndrome by WHO (2021), consumers have no self-control problems at the beginning of SSB consumption but may gradually increase the SSB intake to maintain the same level of utility due to the tolerance to added sugar in the process of periodical consumption in a long term. Once consumers develop sugar dependence, they may feel compulsive to consume SSBs and have difficulties in controlling SSB intake in terms of onset, termination or the amount consumed. In the framework of economics, the addictiveness of SSBs or carbohydrates is manifested in that the past, current and future consumption complement each other according to the rational addiction theory proposed by Becker and Murphy (1988) [7–9]. Given the addictiveness of SSBs, there is a strong probability that SSBs and other addictive substances are interdependent in consumption.

## 3 Empirical strategy

In this study the interdependence between SSBs and cigarettes in consumption is based on the premise that SSBs are addictive and that the consumption of addictive substances is supposed to be correlated. In contrast, researchers are initially motivated to explore the correlation between smoking and drinking by the direct observation that smokers account for a high proportion of drinkers [12, 32]. There is a lack of such evidence for SSB and cigarette consumption making the interdependence between SSBs and cigarettes not as explicit as that between cigarettes and alcohol. Therefore, we first approach the data in a non-parametric method by analyzing the smoking behaviors of SSB consumers to get a general idea about the potential interdependence and then examine such interdependence empirically.

Technically, the standard approach to investigate the interdependence between the consumption of cigarettes and alcohol is to calculate cross-price elasticities from price and income coefficient estimates with demand functions [10, 12, 14, 16, 17, 19, 20]. However, such method is often seriously hindered by the lack of individual-level price information or limited price variation [21]. Therefore, including smoking-relevant variables, such as smoking or not [33, 34] and the amount of cigarettes smoked [21, 22], into the alcohol consumption equation is employed to explore the correlation through the coefficient estimates. Besides, because the stimulators giving rise to the interdependence are unclear and therefore included in the error terms of demand equations, the correlation between the error terms in cigarette and alcohol equations for each individual provides indirect evidence for the interdependence between the consumption of the two goods. In light of this view, Yen (2005) observes significant error correlations between the participation equation and the corresponding quantity equation for cigarettes or alcohol, and between the participation or quantity equations for cigarettes and alcohol with a multivariate sample selection model. However, the lack of price data limits the scope of analysis.

Therefore, we build our work upon Yen (2005). We first apply Heckman sample selection model to examine the participation and quantity decisions for SSB consumption and include smoking-relevant variables into each equation. Specifically, the consumption frequency of SSBs is applied as the proxy of quantity decisions. In general, the more frequently one consumes SSBs, the higher the probability of taking in excessive added sugar and the higher the risk of SSB consumption is. Consumption frequency is an effective indicator to identify the health risks of SSB consumption. Furthermore, Liebman et al. (2003) found that individuals consumed SSBs once a week and more are more likely to be overweight or obese than those who consumed less than once a week. De et al. (2011) pointed out that men who consumed 4.5–7.6 servings weekly exhibited increased risk of developing type 2 diabetes compared to those who did not consume SSBs. A study conducted in the United States concluded that if young children aged 10–12 months consumed SSBs more than three times per week, they were 83% more likely to develop dental caries five years later [37]. After controlling confounding factors, Ma et al. (2016) suggested that the possibility of consumers consuming SSBs three times or more weekly in developing pre-diabetes was approximately 46% higher than consumers in the lowest frequency group. We thus classify individuals who consumed SSBs 3–4 times a week or more as high-frequent consumers [35–38]. Smoking-relevant variables include ever-smoked, currently smoking and the amount of cigarettes smokes daily. Correspondingly the whole sample is divided into ever-smoked group and currently smoking group to further explore the group difference of cross effects.

Series of robustness tests of the correlations are conducted. First, we replace the consumption frequency with SSB weekly intake per capita as the proxy of quantity decisions in the sample selection model. Second, according to Wang et al. (2018), approximately 47% and 3% of men and women smoked in the period from 2003–2013 in China [39]. Because the smoking population is predominantly male, we then narrow our research subjects to male respondents to further examine the interdependence. Third, given that the consumption of SSBs and cigarettes are probably simultaneous decisions, we model the demand for SSBs and cigarettes in system methods, adopting seemingly unrelated bivariate probit or seemingly unrelated regression, to validate the correlation captured in the sample selection model through the estimates of error correlation and cross price effects. The multiple equation method allowing for the error correlation is more statistically efficient than single equation method [18], and more behaviorally appealing in comparison with the structural equation model employed by Tauchmann et al. (2012) and Ren et al. (2020) which solely focuses on the correlation between the amount of cigarette smoked and alcohol intake.

## 4 Data and method

### 4.1 CHNS

The CHNS is an international collaborative project between the National Institute for Nutrition and Health at the Chinese Center for Disease Control and Prevention and the Carolina Population Center at the University of North Carolina [40]. It has surveyed individuals, households and communities from nine provinces (Liaoning, Hei Longjiang, Shandong, Henan, Hubei, Hunan, Guangxi, Guizhou, Jiangsu) across China every 2 to 4 years to track changes in the health, nutrition, population, and economic status of Chinese residents Since 1989 [41]. Three more provinces were added in the 2015 round, consisting of Shanxi, Zhejiang and Yunnan. The CHNS obtains samples with a multistage cluster design. The counties within each province are first stratified by income and a weighted sample of four counties is selected. In each county or city, communities and households in each community are randomly selected. All members of each household are interviewed. Because of the randomness of the sample selection in each round of survey, the data is strongly unbalanced [22]. However, as the survey covers almost 56% of the population of China from urban and rural areas [41], it is still a good representative of the Chinese population. Extensive literature has applied CHNS to explore how public policies or programs implemented by governments impact the health and nutritional status of the Chinese so far [40]. More importantly, the beverage sales in China have increased rapidly since 2000 [29], thus the CHNS during this period could dynamically reflect the changes of Chinese SSB consumption. Other research focusing on the SSB consumption of Chinese usually conducted small-scale surveys in urban areas or utilized sales data of urban populations from certain provinces, making the results lack representativeness [42, 43].

The CHNS has asked all the adult respondents to report their SSB consumption since 2004. We first identify SSB consumers according to individuals’ responses to the question “Did you drink soft drinks or sugared fruit drinks in the last year?”. Individuals who consumed SSBs last year were further asked about their consumption frequency, including every day, 3–4 times a week, 1–2 times a week, 1–2 times a month, no more than once a month, or unknown. Individuals consuming SSBs 3–4 times a week or more are categorized as high-frequent consumers (Section 3). Those who consume SSBs 1–2 times a week or less are low-frequent consumers correspondingly. Although individuals were also asked to report their weekly SSB intake with the question “Do you drink this beverage and how much do you drink in each week (liters)?” in CHNS, a large number of individuals’ responses are missing. To calculate the quantity of SSB intake, we follow the measurement adopted by Li (2019) and multiply the self-reported SSB consumption frequency by the standard volume of one serving of beverage. The standard SSB bottle volume is set at 355ml which was applied in the community survey of CHNS to collect price data of SSBs. The consumption frequency every day, 3–4 times a week, 1–2 times a week, 1–2 times a month, and no more than once a month are transformed to 7 times a week, 3.5 times a week, 1.5 times a week, 0.35 times a week, and 0.25 times a week correspondingly. Specifically, if one consumes SSBs every day, the average weekly intake is 2485ml. Given that the survey data in 2015 regarding SSB consumption is unavailable, we construct an unbalanced panel dataset using four rounds of CHNS from 2004 to 2011.

As for cigarette consumption, the survey has asked all the adult respondents an array of smoking-related questions since 1991, “Have you ever smoked?”, “Do you still smoke cigarettes now” and “How many cigarettes do you smoke per day?” Respondents who have smoked cigarettes but do not smoke now are categorized as former smokers. Those who have smoked cigarettes and still smoke now are current smokers. Accordingly, the ever-smoked subgroup includes former and current smokers while the currently smoking subgroup includes current smokers only. The cigarettes smoked daily by current smokers are also collected.

In the Heckman sample selection model, the dependent variable in the participation equation of SSB consumption is whether one is a SSB consumer while in the quantity equation is whether one is a high-frequent consumer or the amount of SSB weekly intake. Smoking-relevant variables are included as key variables but are dependent variables in cigarette demand equations in the simultaneous equation models. Other explanatory variables include price, income, community food accessibility, gender, age, age^2^, education, urban, province and year given previous research [18, 20, 22].

Prices play a critical role in consumption decisions. However, in CHNS only retail prices for representative foreign or domestic brands of beverages and cigarettes from the free market in the community survey are available. Such community-level price information has been criticized for lack of variation [22]. As the individual-level price data and actual expenditure information are unavailable, we still incorporate the community-level retail prices as the proxies of SSB and cigarette price into demand equations to avoid omitted variables bias. For SSBs, the domestic brand provided in the 2004 and 2009 surveys is Jianlibao. It is Wahaha and Feichang kele in 2006 and described as “soft drink similar to Coca Cola" in 2011. The domestic brand varies across the four round surveys but the foreign brand provided is Coca Cola, remaining unchanged since 2004. Thus we use the price of a standard serving (355ml) of Coca Cola from the free market to measure SSB price given the popularity of Coca Cola in China. For cigarette, the CHNS also collected the price data for four categories of cigarettes consisting of the most expensive cigarettes, Hong Ta Shan, Marlboro cigarettes, and local commonly smoked cigarettes. We use the price of a standard pack (20 cigarettes) for local commonly smoked cigarettes from free market to measure cigarette price in view of the popularity of the local commonly smoked cigarettes. The price data from free markets is exogenous, avoiding the problem of endogeneity arising from the self-reported prices which may reflect individual preference to some extent. Additionally, the prices in the community unit concerning a certain brand of beverage or cigarettes may also serve as the proxies for price differentials across regions and years [18, 31].

Individual food choices are constrained by household income in China [44, 45]. We use total household income and divide it by household size to generate average income per capita, which is also one of the most commonly controlled variables in cigarette consumption [18]. Food accessibility, measuring the number or density of stores supplying the target goods in a given areas or the distance from the community to typical markets, reflects the degree of convenience for one to access the target foods and plays an important role in affecting individuals’ food choices, especially for unhealthy foods [46–48]. We then control the community food accessibility in the SSB demand equation using the modern market score, the secondary indicator of the community urbanization index provided by CHNS [49]. It counts the number of supermarkets, cafes, indoor restaurants etc. within the community boundary and then evaluates the food accessibility for each community on a scale of 0 to 10.

There is a remarkable gender difference in cigarette consumption as aforementioned. More than 93% of smokers are male in our sample (Table 2). Relatively, 42.29% of SSB consumers are male while for high-frequent consumers the percentage is 54.68%. In contrast, gender plays a role in SSB consumption different from that in cigarette consumption. Age and Urban are relevant to SSB consumption as previous studies point out that individuals from the 18–44 age group and urban areas take in the most added sugar through SSB consumption [50] and are also strong predictors for cigarette consumption [18]. Individuals with better education are supposed to be more aware of the adverse effects on the healthiness of SSB and cigarette consumption than others [18]. However, Tauchmann et al. (2012) argued that income and education should be excluded given the potential endogeneity. Following Ren et al. (2020), we control income and education to avoid unobserved heterogeneity but interpret the coefficients with cautions. Other variables such as working status, the number of children etc. are not included out of concern for the potential endogeneity [21, 22].

### 4.2 Summary statistics

We accessed the dataset from CHNS on September 22, 2022 and had no access to information that could identify individual participants. After removing respondents who are unknown about their SSB or cigarette consumption and missing and extreme values, a total of 32187 respondents are obtained eventually with 9768 SSB consumers, 1099 high-frequent consumers, 10338 former and current smokers, and 9095 current smokers. Table 1 presents variables relevant to SSB and cigarette consumption in our study. Fig 1 shows the SSB and cigarette consumption from 2004 to 2011. Overall, the proportion of individuals who consumed SSBs in the whole sample increased from 25.58% in 2004 to 38.16% in 2011, in tune with the rapid increase in beverage sales in China after 2000 [29]. Especially, the proportion of high-frequent consumers in the sample of SSB consumers fluctuates in the range of 9.7% and 12.31% from 2004 to 2011. In light of the large population of China, high-frequent SSB consumers are not a minority and the excessive intake of SSBs induced by high-frequent consumption deserves more attention. As for cigarette consumption, the proportion of individuals who have smoked in the whole sample falls slightly from 33.55% in 2004 to 30.93% in 2011 and that of those giving up smoking ranges from 10% to 14% correspondingly, which is consistent with existing research [51].

**Table 1:** SSB and cigarette consumption from 2004 to 2011.

Variables	Definition		2004	2006	2009	2011	The Whole sample
**SSB consumption**							
SSB consumer	Having consumed SSBs last year (1/0)		25.57	23.2	34.93	38.15	30.35
High-frequent consumer	Consuming SSBs 3–4 times per week or more (1/0)		12.25	11.63	9.7	11.75	11.25
SSB intake	The amount in milliliter of SSB intake per week per capita (frequency * 355ml)	SSB consumer	413.35 (569.51)	405.70 (524.09)	348.66 (489.30)	376.81 (526.12)	382.22 (525.59)
		High-frequent consumer	1741.50 (610.33)	1642.07 (581.64)	1631.94 (577.46)	1627.5 (575.39)	1657.42 (586.25)
**Cigarette consumption**							
Ever-smoked	Having ever smoked cigarettes (1/0)		33.59	32.36	31.58	30.92	32.12
Currently smoking	Still smoking cigarettes (1/0)		29.85	27.89	28.31	27.00	28.26
Amount smoked	The amount of cigarettes smoked per day per capita		15.10 (6.93)	15.17 (7.12)	15.14 (7.19)	15.18 (7.02)	15.15 (7.06)

Data source: CHNS (2004–2011) Note: The mean values are provided for continuous variables with standard deviation in parentheses; the percentages for discrete variables are provided.

As for the weekly average intake of SSBs per capita, there is a high probability for high-frequent consumers to take in excessive added sugars. Individuals in the whole sample consume 382.22ml of SSBs on average per week while that for high-frequent consumers reaches as high as 1657.42ml (Table 1). The sugar content of a bottle of regular Coke cola is 11.1g per 100ml in China. Accordingly, the high-frequent consumers may take in 178.67g of added sugar per week through SSBs alone, with an average daily intake of 25.52g. However, the Dietary Guidelines for Chinese Residents (2022) recommends that the intake of added sugar should be no more than 50g per day, preferably below 25g. High-frequent SSB consumers distinguish themselves from general consumers in terms of health risks. Regarding cigarette consumption, the average amount of cigarettes smoked daily per capita is around 15.2 in the whole sample. Different from SSB consumption, even low cigarette consumption would increase individual health risks significantly [52] and thus WHO (2003, 2021, 2023) strongly recommends quitting smoking instead of reducing daily cigarettes smoked [1, 3, 53]. In other words, regardless of the amount of cigarettes smoked daily, the health risks of current smokers are much higher than those of former smokers, let alone non-smokers. Notably, the categorization of the subgroups for SSB and cigarette consumption in our study is corresponding to the degree of health risks.

Table 2 presents the summary statistics for different subgroups. The price for SSBs and cigarettes and average income per capita are adjusted with the 2011 retail price index and consumer price index respectively. In the whole sample, the average price adjusted is 3.09 yuan per 355ml for SSBs and 5.58 yuan per pack for cigarettes. The average price faced by high-frequent consumers are the lowest in comparison with other subgroups. The score of community food accessibility is 4.68 for SSBs and high-frequent consumers reside in communities with the highest level of food accessibility. 47.87% of the respondents is male with an average age of 48.82 and 35.31% are from urban area. The average income per capita adjusted is 10323.27 yuan. Regarding the gender difference in cigarette consumption, male accounts for 93.10% of the sample of current smokers, consistent with the finding of Wang et al. (2018) that 47.2% of male smoked while that for female is 2.7% in 2013 [39].

**Table 2:** Summary statistics.

Variables	Definition	The whole sample	SSB consumer	High-frequent consumer	Ever-smoked	currently smoking	Male		
							The whole sample	Ever-smoked	currently smoking
Price_SSBs_	The adjusted community price for coke cola (Yuan/355ml per bottle)	3.093	3.094	2.896	3.075	3.079	3.099	3.085	3.087
		(1.243)	(1.236)	(1.084)	(1.168)	(1.165)	(1.250)	(1.084)	(1.179)
Price_Cigars_	The adjusted community price for commonly smoked cigarettes (Yuan/20 cigarettes per pack)	5.578	5.518	5.578	5.566	5.440	5.605	5.561	5.430
		(7.401)	(6.574)	(4.360)	(7.361)	(6.865)	(7.436)	(7.256)	(6.723)
Average income per capita	The adjusted average household total income of family member (yuan)	10323.27	11469.09	13374.34	10330.56	10077.4	10559.63	10377.7	10106.54
		(9396.37)	(9865.01)	(10656.96)	(9514.64)	(9313.15)	(9502.82)	(9540.98)	(9317.66)
Community Food accessibility	The score based on the number of food or beverage store in community (0–10)	4.680	4.916	5.392	4.529	4.459	4.671	4.589	4.521
		(2.923)	(2.936)	(2.811)	(2.913)	(2.901)	(2.921)	(2.918)	(2.906)
Male (%)	Male (1/0)	47.87	42.48	55.69	93.22	93.10			
Age	Age in years	48.82	42.54	36.526	49.814	48.67	48.619	49.116	47.885
		(15.094)	(14.998)	(14.964)	(14.406)	(14.056)	(15.132)	(14.276)	(13.845)
Age^2^	Age * Age	2611.37	2034.96	1557.86	2688.94	2566.05	2592.79	2616.18	2484.61
		(1508.30)	(1380.61)	(1313.83)	(1461.43)	(1402.34)	(1502.49)	(1435.09)	(1364.55)
Education (%)	Senior high school and below (base)	74.28	67.08	54.78	74.48	74.70	69.92	73.11	73.27
	Vocational school, colleges and universities	20	24.46	31.76	20.64	20.64	23.07	21.74	21.77
	Master and above	5.72	8.47	13.47	4.88	4.66	7.01	5.15	4.96
Urban (%)	Survey area, urban or rural (1/0)	35.31	40.23	50.86	35.11	34.17	35.27	35.03	34.09

Data source: CHNS (2004–2011) Note: Standard Deviation in parentheses

### 4.3 Heckman’s sample selection model

To empirically explore the interdependence between SSB and Cigarette consumption, we separate the consumption decision of SSBs into a two-step process. In the first stage, individuals decide whether or not to consume SSBs, namely the participation decision. If one decides to consume SSBs, the next is to decide how much SSBs he or she wants to take in, namely the quantity decision. Therefore, the dependent variable in the participation equation of SSBs is whether one has consumed SSBs and that in the quantity equation is whether one is a high-frequent consumer or the amount in milliliter of SSB intake per week per capita. A closer look at the dataset reveals that there is a large number of zero observations, accounting for approximately 70% of SSB consumption in the whole sample. Given the censored dependent variables, the Heckman sample selection model is used to estimate the two-step decision equations for SSBs.

In technical terms, the Heckman sample selection model accommodates the correlation between error terms of participation and quantity equations. It assumes the dominance of participation decision, meaning that the zero consumption of SSBs results from the participation decision rather than the quantity decision. When the participation and quantity equations are independent, the sample selection model is then reduced to two separate equations [54]. A preliminary understanding of the interdependence between SSB and cigarette consumption would be drawn from the coefficient estimates of smoking-relevant variables from the sample selection model, which can be written as:

$$\begin{array}{ll} Y_{SSBs} & =\beta_{0}+\beta_{1}*Price_{SSBs}+\beta_{2}*X_{Cigars}+\sum \alpha *X_{1}+\mu_{i}+year_{t}+\\ & prov_{k}+\varepsilon \;\left(\text{t}=1,2,3,4;\;\text{k}=1,\ldots ,9\right) \end{array}$$ (1)

Where *Y*_*SSBs*_ denotes whether one consumes SSBs in the participation equation, and represents whether one is a high-frequent SSB consumer or the average SSB intakes per week per capita in the quantity equation. *Price*_*SSBs*_ is the adjusted community price for cola. *X*_*Cigars*_ represents smoking-relevant variables, including whether one has smoked before, whether one is currently smoking, or the number of cigarettes consumed per day per capita and *β*_2_ reflects the interdependence concerned. *X*_1_ denotes covariates related to SSB consumption and demographic characteristics. For a panel dataset, *μ*_*i*_ controls the individual specific effect. When *μ*_*i*_ is assumed to be independent, it is a random effect model. If *μ*_*i*_ is fixed, a fixed effect model should be estimated. *year*_*t*_(t indicates the 4 round surveys) and *prov*_*k*_ (k indicates the 9 provinces surveyed) are dummy variables used to capture time and region fixed effects. ε is the error term.

### 4.4 Simultaneous equation model

Based on the results of sample selection model, we then applied the simultaneous equation method (SEM), the seemingly unrelated bivariate probit (SUBP) or the seemingly unrelated regression (SUR), to further validate the correlations. Given the extant hypotheses or theories aforementioned, the error terms in one’s demand equations for SSBs and cigarettes are supposed to be correlated due to unexplained individual characteristics. SUBP and SUR models accommodate such error correlations and can be written as:

$$Y_{SSBs}=\beta_{0}+\beta_{1}*\;Price_{SSBs}+\beta_{2}*\;Price_{Cigars}+\;\sum \theta *X_{1}+\;year_{t}+prov_{k}+\varepsilon$$

$$\begin{array}{ll} Y_{Cigars} & =\delta_{0}+\delta_{1}*\;Price_{SSBs}+\delta_{2}*\;Price_{Cigars}++\sum \varphi *X_{2}+\;year_{t}+\\ & prov_{k}+\varepsilon \;\left(\text{t}=1,2,3,4;\;\text{k}=1,\ldots ,9\right) \end{array}$$ (2)

When *Y*_*SSBs*_ denotes whether one consumes SSBs or is a high-frequent SSB consumer and *Y*_*Cigars*_ represents whether one has smoked or is smoking currently, *Y*_*SSBs*_ and *Y*_*Cigars*_ are both dummy variables and therefore Eq (2) is a SUBP model. When *Y*_*SSBs*_ and *Y*_*Cigars*_ denote the average SSB intakes per week per capita and the number of cigarettes consumed per day per capita respectively, Eq (2) is a SUR model. *Price*_*SSBs*_ and *Price*_*Cigars*_ are the adjusted community prices for cola and regular cigarettes and *β*_2_ and *δ*_1_ are the cross price effects. *X*_1_ or *X*_2_ represents covariates related to SSB or cigarette consumption and demographic characteristics.

## 5 Results and analysis

### 5.1 Non-parametric analysis

**Fig 2:**
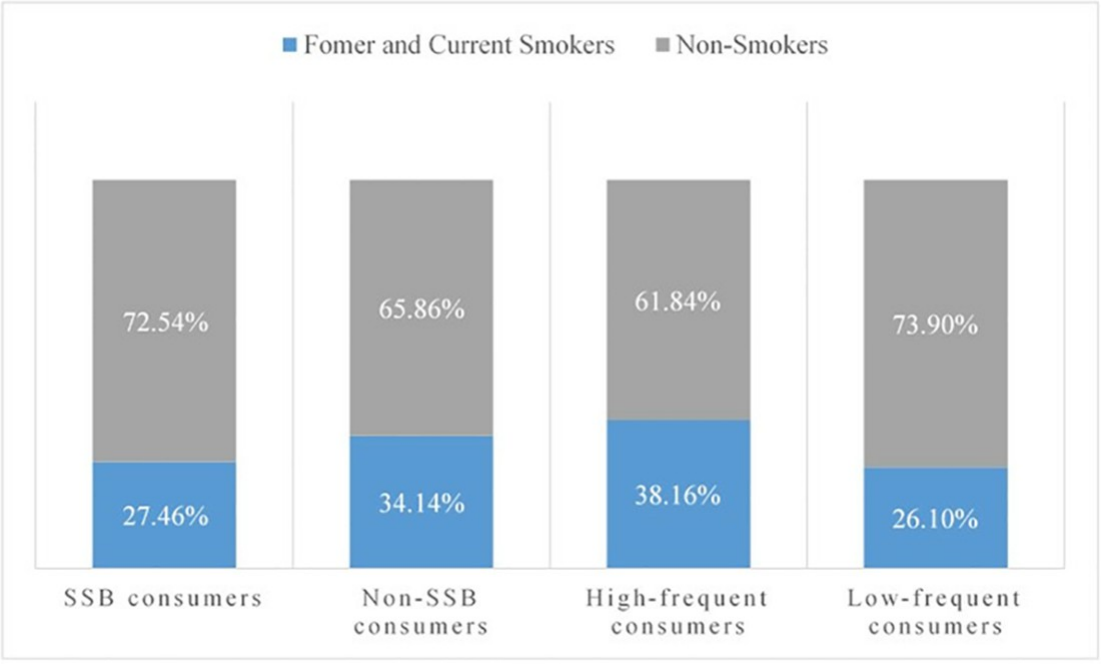
The cigarette consumption in SSB consumers.

Fig 2 presents the cigarette consumption in SSB consumers. The proportion of former and current smokers in the group of SSB consumers is 27.46%, lower than that in the group of non-SSB consumers, which is 34.14%. However, such proportion in the group of high-frequent SSB consumers is 38.16%, higher than that in the group of low-frequent SSB consumers, which is 26.10%. Individuals who have smoked are less likely to consume SSBs but possess a higher possibility to be high-frequent SSB consumers, which implies not only the latent correlation between SSB and cigarette consumption but also the group difference of such correlation. Then, we apply Heckman sample selection model to empirically examine the correlations.

### 5.2 Results for Heckman’s sample selection model

Tables 3–5 present the results of the two-step estimation of Heckman sample selection model with consumption frequency denoting quantity decisions in the second stage among the whole sample, the subgroup of ever-smoked and the subgroup of currently smoking respectively. Results for the pooled model without individual effects are provided alongside those for random effect and fixed effect model. Notably, the estimation of the fixed effect models results in a significant reduction in sample size, leading to a certain loss of efficiency. The estimates of inverse mills ratio are statistically significant for the whole sample and the ever-smoked subgroup, justifying the application of the sample selection model. In contrast, no significant error correlation is captured in the subgroup of currently smoking, indicating the group difference.

**Table 3:** The results for Heckman sample selection model in the whole sample.

Variables	Pooled		Random effect		Fixed effect		Male sample					
							Pooled		Random effect		Fixed effect	
	First stage	Second stage	First stage	Second stage	First stage	Second stage	First stage	Second stage	First stage	Second stage	First stage	Second stage
	SSB consumer	High-frequent consumer	SSB consumer	High-frequent consumer	SSB consumer	High-frequent consumer	SSB consumer	High-frequent consumer	SSB consumer	High-frequent consumer	SSB consumer	High-frequent consumer
Ever-smoked	0.029	0.055***	0.046*	0.610***	0.516***	0.733**	0.035	0.072***	0.058*	0.717***	0.555***	0.536*
	(0.022)	(0.010)	(0.027)	(0.112)	(0.065)	(0.300)	(0.024)	(0.015)	(0.031)	(0.133)	(0.071)	(0.320)
Log(Price_SSBs_)	-0.116***	-0.040***	-0.101***	-0.396**	0.098	-0.324	-0.069	-0.039	-0.042	-0.189	0.123	-0.228
	(0.029)	(0.014)	(0.033)	(0.163)	(0.067)	(0.350)	(0.042)	(0.026)	(0.049)	(0.209)	(0.103)	(0.491)
Log(Average income per capita)	0.062***	0.014***	0.064***	0.121**	0.072***	-0.107	0.057***	0.034***	0.057***	0.247***	0.082***	-0.055
	(0.007)	(0.005)	(0.008)	(0.056)	(0.014)	(0.070)	(0.010)	(0.009)	(0.012)	(0.078)	(0.022)	(0.103)
Community Food accessibility	0.005*	0.006***	0.006*	0.077***	0.042***	-0.038	0.003	0.008***	0.004	0.083***	0.063***	-0.005
	(0.003)	(0.001)	(0.004)	(0.017)	(0.009)	(0.045)	(0.004)	(0.003)	(0.006)	(0.023)	(0.013)	(0.064)
Male	-0.261***	-0.012	-0.308***	0.012								
	(0.021)	(0.018)	(0.026)	(0.179)								
Age	-0.069***	-0.022***	-0.078***	-0.200***	0.188**	-0.927**	-0.084***	-0.041***	-0.099***	-0.274***	0.311***	-0.823
	(0.003)	(0.004)	(0.004)	(0.037)	(0.073)	(0.414)	(0.004)	(0.009)	(0.006)	(0.065)	(0.109)	(0.585)
Age^2^	0.0004***	0.0002***	0.0005***	0.001***	-0.0003	-0.001	0.001***	0.0003***	0.001***	0.002***	-0.002***	-0.002
	(0.00003)	(0.00002)	(0.00004)	(0.0002)	(0.0002)	(0.001)	(0.00004)	(0.0001)	(0.0001)	(0.0005)	(0.0003)	(0.001)
Education												
Vocational schools, colleges and universities	0.117***	0.031***	0.135***	0.296***	0.151**	0.523	0.116***	0.050**	0.129***	0.292*	-0.397***	0.455
	(0.021)	(0.011)	(0.026)	(0.115)	(0.077)	(0.326)	(0.029)	(0.020)	(0.037)	(0.156)	(0.106)	(0.460)
Master and above	0.211***	0.045***	0.244***	0.407**	-0.353**	0.866*	0.201***	0.070**	0.224***	0.457*	-3.153***	1.219*
	(0.035)	(0.017)	(0.043)	(0.175)	(0.140)	(0.484)	(0.047)	(0.033)	(0.060)	(0.246)	(0.193)	(0.668)
Urban	0.144***	0.051***	0.168***	0.535***			0.131***	0.085***	0.162***	0.684***		
	(0.018)	(0.011)	(0.022)	(0.122)			(0.026)	(0.021)	(0.034)	(0.173)		
Constant	0.928***	0.359***	1.090***	0.056			0.987***	0.281***	1.178***	-0.949		
	(0.098)	(0.049)	(0.120)	(0.534)			(0.144)	(0.089)	(0.181)	(0.745)		
Year FE	Yes	Yes	Yes	Yes	Yes	Yes	Yes	Yes	Yes	Yes	Yes	Yes
Individual FE	No	No	Yes	Yes	Yes	Yes	No	No	Yes	Yes	Yes	Yes
Province FE	Yes	Yes	Yes	Yes	No	No	Yes	Yes	Yes	Yes	No	No
N	32187	9768	32187	9768	12986	1047	15407	4149	15407	4149	5592	533

Note: The random effect model includes the random-effect probit model in the first stage and the random-effect logit model in the second, both of which employ cluster-robust standard errors given the potential error autocorrelation. The fixed effect model comprises fixed-effect probit model in the first stage using jackknife bias corrections for individual and time fixed effects, and the fixed-effect logit model in the second. Standard Deviation in parentheses.

***, ** and * signify statistical significance at 0.01, 0.5, and 0.1level respectively. When the per capita total income of a household is 0, add 1 before taking the logarithm to avoid sample selection bias.

**Table 4:** The results for Heckman sample selection model in the subgroup of ever-smoked.

Variables	Pooled		Random effect		Fixed effect		Male sample					
							Pooled		Random effect		Fixed effect	
	First stage	Second stage	First stage	Second stage	First stage	Second stage	First stage	Second stage	First stage	Second stage	First stage	Second stage
	SSB consumer	High-frequent consumer	SSB consumer	High-frequent consumer	SSB consumer	High-frequent consumer	SSB consumer	High-frequent consumer	SSB consumer	High-frequent consumer	SSB consumer	High-frequent consumer
Currently smoking	-0.194***	-0.062	-0.213***	-0.333	-0.375***	-0.451	-0.183***	-0.056	-0.201***	-0.327	-0.354**	-0.451
	(0.046)	(0.040)	(0.054)	(0.308)	(0.137)	(0.533)	(0.047)	(0.040)	(0.057)	(0.308)	(0.140)	(0.534)
Log(Price_SSBs_)	-0.068	-0.047	-0.032	-0.282	0.681***	-0.315	-0.067	-0.045	-0.034	-0.249	0.714***	-0.325
	(0.053)	(0.033)	(0.061)	(0.262)	(0.137)	(0.609)	(0.054)	(0.033)	(0.063)	(0.265)	(0.140)	(0.610)
Log(Average income per capita)	0.061***	0.032***	0.063***	0.228**	0.187***	-0.121	0.065***	0.031**	0.068***	0.216**	0.202***	-0.120
	(0.012)	(0.012)	(0.015)	(0.098)	(0.029)	(0.164)	(0.013)	(0.013)	(0.015)	(0.099)	(0.029)	(0.164)
Community Food accessibility	-0.002	0.008**	-0.003	0.090***	0.040**	0.006	0.0002	0.009***	-0.001	0.095***	0.044**	0.010
	(0.006)	(0.003)	(0.007)	(0.027)	(0.017)	(0.084)	(0.006)	(0.003)	(0.007)	(0.028)	(0.017)	(0.085)
Male	-0.283***	-0.049	-0.334***	0.038								
	(0.062)	(0.057)	(0.076)	(0.482)								
Age	-0.086***	-0.044***	-0.099***	-0.280***	0.226	-1.166	-0.087***	-0.043***	-0.101***	-0.274***	0.237	-1.162
	(0.006)	(0.012)	(0.007)	(0.091)	(0.148)	(0.765)	(0.006)	(0.013)	(0.008)	(0.091)	(0.153)	(0.764)
Age^2^	0.001***	0.0003***	0.001***	0.002***	-0.002***	-0.002	0.001***	0.0003***	0.001***	0.002***	-0.002***	-0.002
	(0.0001)	(0.0001)	(0.0001)	(0.001)	(0.0004)	(0.002)	(0.0001)	(0.0001)	(0.0001)	(0.001)	(0.0004)	(0.002)
Education												
Vocational schools, colleges and universities	0.093**	0.045*	0.104**	0.251	-0.397***	0.569	0.090**	0.042*	0.102**	0.227	-0.329**	0.569
	(0.036)	(0.025)	(0.047)	(0.186)	(0.152)	(0.682)	(0.037)	(0.025)	(0.048)	(0.185)	(0.154)	(0.682)
Master and above	0.157**	0.074*	0.166**	0.434	-0.512	2.829**	0.145**	0.073*	0.152*	0.447	-0.558*	2.817**
	(0.067)	(0.043)	(0.084)	(0.298)	(0.327)	(1.386)	(0.068)	(0.043)	(0.086)	(0.297)	(0.328)	(1.389)
Urban	0.088***	0.071***	0.114***	0.593***			0.072**	0.067***	0.095**	0.569***		
	(0.032)	(0.023)	(0.041)	(0.189)			(0.033)	(0.022)	(0.042)	(0.182)		
Constant	1.560***	0.657***	1.815***	1.124			1.236***	0.606***	1.444***	1.185		
	(0.201)	(0.115)	(0.248)	(1.042)			(0.198)	(0.109)	(0.246)	(0.919)		
Year FE	Yes	Yes	Yes	Yes	Yes	Yes	Yes	Yes	Yes	Yes	Yes	Yes
Individual FE	No	No	Yes	Yes	Yes	Yes	No	No	Yes	Yes	Yes	Yes
Province FE	Yes	Yes	Yes	Yes	No	No	Yes	Yes	Yes	Yes	No	No
N	10330	2678	10330	2678	3320	315	9632	2536	9632	2536	3125	310

Note: The random effect model includes the random-effect probit model in the first stage and the random-effect logit model in the second, both of which employ cluster-robust standard errors given the potential error autocorrelation. The fixed effect model comprises fixed-effect probit model in the first stage using jackknife bias corrections for individual and time fixed effects, and the fixed-effect logit model in the second. Standard Deviation in parentheses.

***, ** and * signify statistical significance at 0.01, 0.5, and 0.1level respectively. When the per capita total income of a household is 0, add 1 before taking the logarithm to avoid sample selection bias.

**Table 5:** The results for Heckman sample selection model in the subgroup of currently smoking.

Variables	Pooled		Random effect		Fixed effect		Male sample					
							Pooled		Random effect		Fixed effect	
	First stage	Second stage	First stage	Second stage	First stage	Second stage	First stage	Second stage	First stage	Second stage	First stage	Second stage
	SSB consumer	High-frequent consumer	SSB consumer	High-frequent consumer	SSB consumer	High-frequent consumer	SSB consumer	High-frequent consumer	SSB consumer	High-frequent consumer	SSB consumer	High-frequent consumer
Cigarettes smoked daily per capita	-0.006**	0.002	-0.005*	0.021*	-0.007	0.014	-0.005**	0.002	-0.005	0.021*	-0.010	0.019
	(0.002)	(0.001)	(0.003)	(0.013)	(0.006)	(0.027)	(0.002)	(0.001)	(0.003)	(0.013)	(0.006)	(0.028)
Log(Price_SSBs_)	-0.092	-0.028	-0.066	-0.178	0.396**	-0.759	-0.092	-0.025	-0.067	-0.153	0.404**	-0.757
	(0.059)	(0.032)	(0.065)	(0.295)	(0.156)	(0.712)	(0.060)	(0.033)	(0.067)	(0.301)	(0.159)	(0.715)
Log(Average income per capita)	0.059***	0.019*	0.062***	0.175*	0.224***	-0.190	0.061***	0.017	0.065***	0.158	0.208***	-0.192
	(0.013)	(0.011)	(0.016)	(0.104)	(0.032)	(0.212)	(0.014)	(0.012)	(0.017)	(0.105)	(0.033)	(0.215)
Community Food accessibility	-0.003	0.009***	-0.004	0.103***	0.077***	0.047	0.001	0.009***	-0.0002	0.105***	0.077***	0.047
	(0.006)	(0.003)	(0.007)	(0.031)	(0.019)	(0.105)	(0.006)	(0.003)	(0.008)	(0.031)	(0.020)	(0.106)
Male	-0.234***	-0.002	-0.270***	0.159								
	(0.068)	(0.051)	(0.082)	(0.514)								
Age	-0.088***	-0.028**	-0.102***	-0.198*	0.176	-1.743*	-0.089***	-0.027**	-0.104***	-0.195*	0.224	-1.658*
	(0.007)	(0.012)	(0.008)	(0.106)	(0.169)	(0.946)	(0.007)	(0.012)	(0.009)	(0.106)	(0.176)	(0.960)
Age^2^	0.001***	0.0002**	0.001***	0.001*	-0.002***	0.001	0.001***	0.0002**	0.001***	0.001*	-0.002***	-0.0004
	(0.0001)	(0.0001)	(0.0001)	(0.001)	(0.0004)	(0.003)	(0.0001)	(0.0001)	(0.0001)	(0.001)	(0.0004)	(0.002)
Education												
Vocational schools, colleges and universities	0.097**	0.035	0.103**	0.231	-0.099	0.651	0.101**	0.031	0.107**	0.210	-0.057	0.904
	(0.040)	(0.024)	(0.051)	(0.208)	(0.169)	(0.800)	(0.041)	(0.024)	(0.052)	(0.209)	(0.171)	(0.776)
Master and above	0.113	0.051	0.119	0.348	3.322***	3.050**	0.108	0.050	0.113	0.367	0.199	3.358**
	(0.075)	(0.038)	(0.089)	(0.324)	(0.378)	(1.477)	(0.076)	(0.038)	(0.091)	(0.325)	(0.379)	(1.503)
Urban	0.072**	0.061***	0.093**	0.543***			0.053	0.062***	0.072	0.557***		
	(0.035)	(0.021)	(0.044)	(0.204)			(0.037)	(0.020)	(0.046)	(0.198)		
Constant	1.485***	0.566***	1.726***	0.384			1.214***	0.586***	1.429***	0.688		
	(0.214)	(0.100)	(0.260)	(1.042)			(0.212)	(0.100)	(0.258)	(0.931)		
Year FE	Yes	Yes	Yes	Yes	Yes	Yes	Yes	Yes	Yes	Yes	Yes	Yes
Individual FE	No	No	Yes	Yes	Yes	Yes	No	No	Yes	Yes	Yes	Yes
Province FE	Yes	Yes	Yes	Yes	No	No	Yes	Yes	Yes	Yes	No	No
N	8469	2215	8469	2215	2629	239	7861	2099	7861	2099	2456	234

Note: The random effect model includes the random-effect probit model in the first stage and the random-effect logit model in the second, both of which employ cluster-robust standard errors given the potential error autocorrelation. The fixed effect model comprises fixed-effect probit model in the first stage using jackknife bias corrections for individual and time fixed effects, and the fixed-effect logit model in the second. Standard Deviation in parentheses.

***, ** and * signify statistical significance at 0.01, 0.5, and 0.1level respectively. When the per capita total income of a household is 0, add 1 before taking the logarithm to avoid sample selection bias.

In the whole sample (Table 3), the coefficients of ever-smoked in the first stage vary in magnitude and significance across pooled, random effect and fixed effect models, but the direction of these coefficients remains consistently positive. Especially in the fixed effect model, the coefficient is statistically significant at the 0.01 level, which indicates that individuals who ever smoked incline to consume SSBs. In the second stage, the coefficients are significantly positive across all the three models, albeit with varying magnitudes, implying that individuals who ever smoked possess a higher probability of being a high-frequent consumer. These positive correlations signify the complementarity between SSBs and cigarettes (consistent with the “addictive personality “or “learning-based” hypothesis). The estimation for male consumers only is consistent with that for the whole sample.

In the subgroup of ever-smoked (Table 4), the estimates for currently smoking are significantly negative across pooled, random effect and fixed effect models in the first stage with increasing magnitudes, suggesting that former smokers are more likely to consume SSBs in comparison with current smokers, which reflects substitutability and is supportive of the explanation of “oral drive”[16]. No statistically significant differences are observed between former and current smokers in consumption frequency in the second stage across the three models. The estimates for male consumers remain consistent throughout. In the subgroup of smoking currently (Table 5), the coefficients estimated for the amount of cigarettes smoked daily in the first and second stages exhibit opposite signs. So it is with male consumers. Such asymmetry may arise from the group difference. More specifically, the increase in the amount of cigarettes smoked accompanies a decline in the participation of SSB consumption while for those who still consume SSBs, their consumption frequency increases. Consistent with prior analysis, the average amount of cigarettes smoked daily for high-frequent consumers is 14.82, slightly higher than that for low-frequent consumers, 14.57 (Table 1).

Overall, the estimations pertaining to smoking-related variables across the pooled, random effect, and fixed effect models yield consistent conclusions. However, for other covariates, the estimates for pooled model align with those of random effect but generally differ from those of fixed effect. For example, in the whole sample (Table 3), price increases could significantly reduce the participation and quantity of SSB consumption according to the estimates of pooled and random effect models while the fixed effect model reveals no significant impact of price increases on SSB consumption in both stages. Similarly, for the education level of master and above, the coefficients are positively significant in the first and second stage among the pooled and random effect. But in the fixed effect, the coefficient in the first stage is opposite with that in the second stage. Such remarkable difference in the estimations could also be observed in the subgroup of ever-smoked and currently smoking. One possible explanation is that the decrease in sample size resulting from the estimation of fixed effect compromises the representativeness. This speculation is supported by the consistency between the estimates of pooled and random effect models and prior descriptive statistics presented in Table 2. Besides, fixed effect model fails to estimate the time-invariant variables such as male and urban.

Therefore, according to the results of random effect, price increases reduce the demand for SSBs in participation and quantity in the whole sample. In contrast, individuals in the subgroup of ever-smoked and currently smoking are insensitive to beverage price changes. As male accounts for more than 93% of the subgroup of ever-smoked and smoking currently (Table 2), no significant price effects are observed for male sample. Such findings echo with extant literatures that individuals behave differently in response to price changes due to heterogeneity [23]. This highlights the importance of identifying the target groups to improve the effectiveness of price tools in reducing SSB consumption. An increase in the average household income is associated with higher consumption of SSBs in both participation and quantity across all the three groups, including the male sample. Similarly, higher levels of education contributes to increased SSB consumption in general. Female consumers tend to consume more SSBs while no significant gender differences are observed among high-frequent consumers. As expected, community food accessibility positively influences high-frequent SSB consumption across all groups. Urban residents take in significantly more SSBs than their rural counterparts do. Especially, SSB consumption exhibits a U-shaped correlation with age and most samples distribute at the left of the turning point, indicating that older individuals exhibit a reduced demand for SSBs, consistent with the findings of Li (2014).

Tables 6–8 present the results of the two-step estimation of Heckman sample selection model with SSB weekly intake per capita measuring quantity decisions in the second stage among the whole sample, the subgroup of ever-smoked and the subgroup of currently smoking respectively. Results for the pooled, random effect and fixed effect are provided together. Similarly, the estimation of fixed effect lead to a smaller sample and therefore a loss of efficiency. No significant changes are observed in the correlations between error terms, the coefficient estimates for smoking-relevant variables and other covariates in general given that the intake of SSBs weekly is transformed from the consumption frequency. One exception is that in the subgroup of smoking currently the amount of SSB intake positively correlates with the amount of cigarettes smoked across the pooled, random effect and fixed effect models while no significant positive effect is observed in the previous estimation (Table 8). This may because the consumption frequency lacks variation in comparison with the amount of SSB intake. Table 9 summarizes the correlations between SSB and cigarette consumption across different subgroups.

**Table 6:** The results for Heckman sample selection model in the whole sample.

Variables	Pooled		Random effect		Fixed effect		Male sample					
							Pooled		Random effect		Fixed effect	
	First stage	Second stage	First stage	Second stage	First stage	Second stage	First stage	Second stage	First stage	Second stage	First stage	Second stage
	SSB consumer	SSB intake	SSB consumer	SSB intake	SSB consumer	SSB intake	SSB consumer	SSB intake	SSB consumer	SSB intake	SSB consumer	SSB intake
Ever-smoked	0.029	84.236***	0.046*	84.505***	0.516***	117.079***	0.035	118.323***	0.058*	122.496***	0.555***	106.483**
	(0.022)	(16.150)	(0.027)	(16.581)	(0.065)	(42.297)	(0.024)	(23.631)	(0.031)	(20.174)	(0.071)	(47.220)
Log(Price_SSBs_)	-0.116***	-70.133***	-0.101***	-61.671***	0.098	-18.906	-0.069	-56.094	-0.042	-35.359	0.123	34.411
	(0.029)	(23.208)	(0.033)	(19.449)	(0.067)	(32.606)	(0.042)	(41.799)	(0.049)	(30.389)	(0.103)	(58.788)
Log(Average income per capita)	0.062***	31.203***	0.064***	26.714***	0.072***	-6.985	0.057***	60.862***	0.057***	51.405***	0.082***	-5.699
	(0.007)	(7.825)	(0.008)	(7.499)	(0.014)	(10.792)	(0.010)	(14.033)	(0.012)	(11.588)	(0.022)	(19.920)
Community Food accessibility	0.005*	9.837***	0.006*	9.880***	0.042***	2.060	0.003	11.950***	0.004	12.290***	0.063***	4.753
	(0.003)	(2.182)	(0.004)	(2.211)	(0.009)	(5.955)	(0.004)	(4.232)	(0.006)	(3.589)	(0.013)	(10.664)
Male	-0.261***	-9.635	-0.308***	-5.356								
	(0.021)	(28.751)	(0.026)	(28.256)								
Age	-0.069***	-37.681***	-0.078***	-35.985***	0.188**	-64.079	-0.084***	-67.065***	-0.099***	-62.607***	0.311***	-48.167
	(0.003)	(6.134)	(0.004)	(6.028)	(0.073)	(43.097)	(0.004)	(14.164)	(0.006)	(13.132)	(0.109)	(83.512)
Age^2^	0.0004***	0.274***	0.0005***	0.263***	-0.0003	-0.127	0.001***	0.471***	0.001***	0.436***	-0.002***	-0.227
	(0.00003)	(0.039)	(0.00004)	(0.038)	(0.0002)	(0.127)	(0.0006)	(0.100)	(0.0001)	(0.092)	(0.0003)	(0.204)
Education												
vocational schools, colleges and universities	0.117***	45.858***	0.135***	45.692***	0.151**	95.218**	0.116***	63.288*	0.129***	55.633**	-0.397***	75.703
	(0.021)	(17.431)	(0.026)	(17.158)	(0.077)	(46.457)	(0.029)	(32.907)	(0.037)	(27.252)	(0.106)	(70.507)
Master and above	0.211***	90.566***	0.244***	91.317***	-0.353**	172.361**	0.201***	127.087**	0.224***	121.819**	-3.153***	368.537***
	(0.035)	(27.879)	(0.043)	(29.641)	(0.140)	(85.076)	(0.047)	(53.221)	(0.060)	(47.869)	(0.193)	(130.105)
Urban	0.144***	100.938***	0.168***	96.714***			0.131***	148.210***	0.162***	145.693***		
	(0.018)	(18.344)	(0.022)	(18.696)			(0.026)	(33.433)	(0.034)	(31.811)		
Constant	0.928***	812.875***	1.090***	862.535***		3143.605*	0.987***	735.243***	1.178***	847.938***		2612.625
	(0.098)	(80.217)	(0.120)	(74.770)		(1643.043)	(0.144)	(144.644)	(0.181)	(109.805)		(3141.726)
Year FE	Yes	Yes	Yes	Yes	Yes	Yes	Yes	Yes	Yes	Yes	Yes	Yes
Individual FE	No	No	Yes	Yes	Yes	Yes	No	No	Yes	Yes	Yes	Yes
Province FE	Yes	Yes	Yes	Yes	No	No	Yes	Yes	Yes	Yes	No	No
N	32187	9768	32187	9768	12986	9768	15407	4149	15407	4149	5592	4149

Note: The random effect model includes the random-effect probit model in the first stage and the random-effect linear model in the second, both of which employ cluster-robust standard errors given the potential error autocorrelation. The fixed effect model comprises fixed-effect probit model in the first stage using jackknife bias corrections for individual and time fixed effects, and the fixed-effect linear model with cluster-robust standard errors in the second. Standard Deviation in parentheses.

***, ** and * signify statistical significance at 0.01, 0.5, and 0.1level respectively. When the per capita total income of a household is 0, add 1 before taking the logarithm to avoid sample selection bias.

**Table 7:** The results for Heckman sample selection model in the subgroup of ever-smoked.

Variables	Pooled		Random effect		Fixed effect		Male sample					
							Pooled		Random effect		Fixed effect	
	First stage	Second stage	First stage	Second stage	First stage	Second stage	First stage	Second stage	First stage	Second stage	First stage	Second stage
	SSB consumer	SSB intake	SSB consumer	SSB intake	SSB consumer	SSB intake	SSB consumer	SSB intake	SSB consumer	SSB intake	SSB consumer	SSB intake
Currently smoking	-0.194***	-72.335	-0.213***	-61.121	-0.375***	-116.556	-0.183***	-68.208	-0.201***	-57.307	-0.354**	-122.022
	(0.046)	(62.861)	(0.054)	(60.615)	(0.137)	(117.448)	(0.047)	(63.879)	(0.057)	(61.347)	(0.140)	(122.471)
Log(Price_SSBs_)	-0.068	-48.585	-0.032	-32.772	0.681***	-0.650	-0.067	-51.811	-0.034	-36.433	0.714***	-2.266
	(0.053)	(50.765)	(0.061)	(41.975)	(0.137)	(97.043)	(0.054)	(52.582)	(0.063)	(43.067)	(0.140)	(97.893)
Log(Average income per capita)	0.061***	53.054***	0.063***	47.503***	0.187***	15.051	0.065***	54.619***	0.068***	48.421***	0.202***	16.195
	(0.012)	(18.842)	(0.015)	(16.793)	(0.029)	(19.850)	(0.013)	(20.478)	(0.015)	(17.997)	(0.029)	(20.126)
Community Food accessibility	-0.002	11.280**	-0.003	11.374**	0.040**	9.143	0.0002	12.301**	-0.001	12.285***	0.044**	9.358
	(0.006)	(4.923)	(0.007)	(4.493)	(0.017)	(14.491)	(0.006)	(5.090)	(0.007)	(4.581)	(0.017)	(14.865)
Male	-0.283***	12.334	-0.334***	19.553								
	(0.062)	(90.160)	(0.076)	(78.899)								
Age	-0.086***	-63.644***	-0.099***	-59.683***	0.226	-102.322	-0.087***	-64.605***	-0.101***	-60.222***	0.237	-101.187
	(0.006)	(19.670)	(0.007)	(19.779)	(0.148)	(133.698)	(0.006)	(20.470)	(0.008)	(20.147)	(0.153)	(137.258)
Age^2^	0.001***	0.465***	0.001***	0.433***	-0.002***	-0.223	0.001***	0.471***	0.001***	0.434***	-0.002***	-0.278
	(0.0001)	(0.139)	(0.0001)	(0.137)	(0.0004)	(0.287)	(0.0001)	(0.146)	(0.0001)	(0.141)	(0.0004)	(0.301)
Education												
vocational schools, colleges and universities	0.093**	41.852	0.104**	38.113	-0.397***	33.794	0.090**	41.753	0.102**	38.114	-0.329**	48.329
	(0.036)	(38.581)	(0.047)	(35.839)	(0.152)	(114.629)	(0.037)	(39.586)	(0.048)	(36.200)	(0.154)	(115.679)
Master and above	0.157**	133.633**	0.166**	126.776*	-0.512	460.382**	0.145**	133.594**	0.152*	128.116*	-0.558*	520.170**
	(0.067)	(66.911)	(0.084)	(67.470)	(0.327)	(200.457)	(0.068)	(67.871)	(0.086)	(68.148)	(0.328)	(213.051)
Urban	0.088***	99.942***	0.114***	98.355***			0.072**	104.811***	0.095**	103.601***		
	(0.032)	(35.577)	(0.041)	(37.424)			(0.033)	(34.888)	(0.042)	(35.914)		
Constant	1.560***	1219.212***	1.815***	1266.070***		5024.480	1.236***	1237.146***	1.444***	1296.186***		4996.141
	(0.201)	(177.070)	(0.248)	(186.394)		(5416.013)	(0.198)	(173.417)	(0.246)	(163.856)		(5464.450)
Year FE	Yes	Yes	Yes	Yes	Yes	Yes	Yes	Yes	Yes	Yes	Yes	Yes
Individual FE	No	No	Yes	Yes	Yes	Yes	No	No	Yes	Yes	Yes	Yes
Province FE	Yes	Yes	Yes	Yes	No	No	Yes	Yes	Yes	Yes	No	No
N	10330	2678	10330	2678	3320	2678	9632	2536	9632	2536	3125	2536

Note: The random effect model includes the random-effect probit model in the first stage and the random-effect linear model in the second, both of which employ cluster-robust standard errors given the potential error autocorrelation. The fixed effect model comprises fixed-effect probit model in the first stage using jackknife bias corrections for individual and time fixed effects, and the fixed-effect linear model with cluster-robust standard errors in the second. Standard Deviation in parentheses.

***, ** and * signify statistical significance at 0.01, 0.5, and 0.1level respectively. When the per capita total income of a household is 0, add 1 before taking the logarithm to avoid sample selection bias.

**Table 8:** The results for Heckman sample selection model in the subgroup of currently smoking.

Variables	Pooled		Random effect		Fixed effect		Male sample					
							Pooled		Random effect		Fixed effect	
	First stage	Second stage	First stage	Second stage	First stage	Second stage	First stage	Second stage	First stage	Second stage	First stage	Second stage
	SSB consumer	SSB intake	SSB consumer	SSB intake	SSB consumer	SSB intake	SSB consumer	SSB intake	SSB consumer	SSB intake	SSB consumer	SSB intake
Cigarettes smoked daily per capita	-0.006**	3.997*	-0.005*	4.082**	-0.007	9.493*	-0.005**	4.109*	-0.005	4.217**	-0.010	10.026*
	(0.002)	(2.254)	(0.003)	(2.047)	(0.006)	(5.366)	(0.002)	(2.270)	(0.003)	(2.073)	(0.006)	(5.457)
Log(Price_SSBs_)	-0.092	-13.415	-0.066	-14.466	0.396**	-32.356	-0.092	-17.996	-0.067	-18.834	0.404**	-24.811
	(0.059)	(52.125)	(0.065)	(49.758)	(0.156)	(117.290)	(0.060)	(53.532)	(0.067)	(50.950)	(0.159)	(118.082)
Log(Average income per capita)	0.059***	35.231*	0.062***	35.433**	0.224***	0.606	0.061***	34.531*	0.065***	34.614**	0.208***	2.064
	(0.013)	(18.288)	(0.016)	(16.359)	(0.032)	(19.883)	(0.014)	(19.302)	(0.017)	(16.902)	(0.033)	(20.592)
Community Food accessibility	-0.003	13.879***	-0.004	14.333***	0.077***	8.988	0.001	13.666***	-0.0002	14.216***	0.077***	12.044
	(0.006)	(4.777)	(0.007)	(4.978)	(0.019)	(17.887)	(0.006)	(4.895)	(0.008)	(5.058)	(0.020)	(18.299)
Male	-0.234***	105.454	-0.270***	98.632								
	(0.068)	(83.188)	(0.082)	(74.156)								
Age	-0.088***	-35.529*	-0.102***	-36.767*	0.176	-158.275	-0.089***	-36.135*	-0.104***	-37.169*	0.224	-146.855
	(0.007)	(20.204)	(0.008)	(20.926)	(0.169)	(154.911)	(0.007)	(20.586)	(0.009)	(20.696)	(0.176)	(160.389)
Age^2^	0.001***	0.282*	0.001***	0.290*	-0.002***	0.116	0.001***	0.287*	0.001***	0.292**	-0.002***	-0.124
	(0.0001)	(0.145)	(0.0001)	(0.150)	(0.0004)	(0.339)	(0.0001)	(0.148)	(0.0001)	(0.149)	(0.0004)	(0.346)
Education												
vocational schools, colleges and universities	0.097**	14.178	0.103**	16.210	-0.099	22.919	0.101**	13.511	0.107**	15.398	-0.057	54.604
	(0.040)	(38.512)	(0.051)	(38.222)	(0.169)	(136.090)	(0.041)	(39.870)	(0.052)	(38.781)	(0.171)	(139.670)
Master and above	0.113	83.737	0.119	90.254	3.322***	426.077*	0.108	83.529	0.113	90.627	0.199	517.682**
	(0.075)	(61.494)	(0.089)	(71.959)	(0.378)	(231.690)	(0.076)	(62.459)	(0.091)	(72.322)	(0.379)	(231.207)
Urban	0.072**	95.186***	0.093**	93.763**			0.053	109.057***	0.072	107.505***		
	(0.035)	(33.174)	(0.044)	(37.123)			(0.037)	(32.350)	(0.046)	(36.002)		
Constant	1.485***	1026.672***	1.726***	1041.914***		6407.196	1.214***	1166.165***	1.429***	1173.299***		6229.314
	(0.214)	(160.315)	(0.260)	(173.731)		(6162.421)	(0.212)	(163.933)	(0.258)	(157.713)		(6242.636)
Year FE	Yes	Yes	Yes	Yes	Yes	Yes	Yes	Yes	Yes	Yes	Yes	Yes
Individual FE	No	No	Yes	Yes	Yes	Yes	No	No	Yes	Yes	Yes	Yes
Province FE	Yes	Yes	Yes	Yes	No	No	Yes	Yes	Yes	Yes	No	No
N	8469	2215	8469	2215	2629	2215	7861	2099	7861	2099	2456	2099

Note: The random effect model includes the random-effect probit model in the first stage and the random-effect linear model in the second, both of which employ cluster-robust standard errors given the potential error autocorrelation. The fixed effect model comprises fixed-effect probit model in the first stage using jackknife bias corrections for individual and time fixed effects, and the fixed-effect linear model with cluster-robust standard errors in the second. Standard Deviation in parentheses.

***, ** and * signify statistical significance at 0.01, 0.5, and 0.1level respectively. When the per capita total income of a household is 0, add 1 before taking the logarithm to avoid sample selection bias.

**Table 9:** The correlations between SSBs and cigarettes in consumption across different subgroups.

Subgroups	Variables	SSB consumer	High-frequent SSB consumer	SSB intake per week per capita
The whole sample	Ever-smoked	—	Complement	Complement
Subgroup of ever-smoked	Currently smoking	Substitute	—	—
Subgroup of currently smoking	Amount smoked	—	—	Complement

Note: ‘—’ indicates that the correlation is not all statistically significant in the three models, pooled, random effect and fixed effect.

### 5.3 Results for simultaneous equation models

Based on the coefficient estimates of smoking-relevant variables in prior estimation, we apply the simultaneous equation method and utilize the SUBP or SUR model to capture the correlation of error terms between SSB and cigarette demand equations to further test the correlation between SSB and cigarette consumption. We first estimate the SUBP model with whether one has consumed SSBs and whether one has smoked as dependent variables in SSB and cigarette demand equations respectively in the whole sample. The estimation fails the Wald test meaning that the error term in the SSB demand equation doesn’t correlate with that in the cigarette demand equation. Therefore, there is no difference between the result of estimating the demand equations separately and that of estimating them together in a systematic way. It also means that no significant correlation is observed between the participation decision of SSBs and cigarette consumption, which is consistent with the conclusion drawn from the sample selection model.

We then establish three other SUBP models and a SUR model. In the first SUBP model, the dependent variables in SSB and cigarette demand equations are whether one is a high-frequent SSB consumer and whether one has smoked respectively. In the whole sample, the p-value for the Wald test is 0, rejecting the null hypothesis that the error terms of the two demand equation are independent of each other at the significance level of 1%. In the second SUBP model, the dependent variables are whether one has consumed SSBs and whether one is smoking currently. In the subgroup of individuals who ever smoked, the p-value for the Wald test is 0, identifying significant error correlation. In the third SUBP model, the dependent variables are whether one is a high-frequent SSB consumer and whether one is smoking currently. In the subgroup of ever-smoked, the p-value for the Wald test is 0.5788, denying that the error terms are correlated significantly. In the subgroup of smoking currently, we establish the SUR model with average SSB weekly intakes per capita and cigarettes smoked daily per capita as the dependent variables for SSB and cigarette demand equations respectively. The p-value for the Breusch-Pagan test is 0.0203, rejecting the null hypothesis at the significance level of 5% and implying that the error terms are interdependent. Table 10 presents the results for the SUBP and SUR models passing the test. Especially, the error correlation is positive in the whole sample and negative in the ever-smoked subgroup. The former means that individuals who have smoked tend to consume SSB frequently while the latter indicates that current smokers are less likely to consumer SSBs, consistent with results of sample selection models.

**Table 10:** The results for SUBP and SUR model.

Variables	Subgroup of ever-smoked SUBP		The whole sample SUBP		Subgroup of currently smoking SUR		Male sample					
							Subgroup of ever-smoked SUBP		The whole sample SUBP		Subgroup of currently smoking SUR	
	SSB consumer	currently Smoking	High-frequent SSB consumer	Ever smoked	SSB Intake	Amount smoked	SSB consumer	currently Smoking	High-frequent SSB consumer	Ever smoked	SSB intake	Amount smoked
Log(Price_SSBs_)	-0.055	0.073	-0.143**	0.004	0.191	-0.996*	-0.056	0.042	-0.033	-0.038	-2.794	-0.978*
	(0.054)	(0.073)	(0.071)	(0.069)	(47.796)	(0.552)	(0.056)	(0.075)	(0.098)	(0.081)	(49.185)	(0.560)
Log(Price_Cigars_)	-0.028	-0.103***	0.024	-0.052	-31.810	-0.146	-0.022	-0.092***	-0.029	-0.023	-33.226	-0.136
	(0.028)	(0.033)	(0.031)	(0.036)	(23.416)	(0.271)	(0.030)	(0.034)	(0.043)	(0.039)	(24.541)	(0.279)
Log(Average income per capita)	0.064***	-0.071***	0.026	-0.001	34.384***	0.239*	0.068***	-0.076***	0.070**	0.019	33.099***	0.282**
	(0.013)	(0.019)	(0.019)	(0.016)	(10.783)	(0.125)	(0.014)	(0.020)	(0.030)	(0.019)	(11.195)	(0.128)
Community Food accessibility	-0.002		0.032***		14.748***		0.001		0.035***		14.412***	
	(0.006)		(0.007)		(4.743)		(0.006)		(0.010)		(4.904)	
Male	-0.265***	-0.398***	0.292***	2.382***	139.585**	4.345***						
	(0.065)	(0.089)	(0.037)	(0.049)	(59.228)	(0.690)						
Age	-0.088***	0.013	-0.060***	0.037***	-29.241***	0.560***	-0.089***	0.017*	-0.050***	0.057***	28.671***	0.593***
	(0.006)	(0.009)	(0.006)	(0.008)	(4.628)	(0.054)	(0.007)	(0.009)	(0.009)	(0.008)	(4.921)	(0.056)
Age^2^	0.0006***	-0.0004***	0.0005***	-0.0003***	0.230***	-0.006***	0.001***	-0.0004***	0.0004***	-0.001***	0.225***	-0.006***
	(0.0001)	(0.0001)	(0.0001)	(0.0001)	(0.050)	(0.001)	(0.0001)	(0.0001)	(0.0001)	(0.0001)	(0.054)	(0.001)
Education												
vocational schools, colleges and universities	0.096**	-0.096*	0.067	-0.239***	5.619	-1.436***	0.094**	-0.094*	0.020	-0.252***	3.914	-1.360***
	(0.040)	(0.051)	(0.045)	(0.049)	(30.927)	(0.357)	(0.041)	(0.052)	(0.062)	(0.056)	(31.714)	(0.360)
Master and above	0.163**	-0.139	0.051	-0.568***	71.928	-2.348***	0.149**	-0.088	-0.035	-0.605***	70.660	-2.341***
	(0.074)	(0.091)	(0.067)	(0.082)	(55.676)	(0.643)	(0.075)	(0.093)	(0.094)	(0.086)	(57.098)	(0.649)
Urban	0.097***	-0.107**	0.175***	0.042	91.553***	1.082***	0.080**	-0.117***	0.194***	-0.016	105.681***	-1.168***
	(0.035)	(0.044)	(0.041)	(0.047)	(28.414)	(0.324)	(0.036)	(0.045)	(0.060)	(0.053)	(29.717)	(0.334)
Constant	1.368***	2.678***	0.122	-2.632***	1023.984***	0.008	1.076***	2.249***	-0.167	-0.765***	1196.298***	3.472*
	(0.211)	(0.302)	(0.221)	(0.243)	(157.939)	(1.838)	(0.209)	(0.304)	(0.331)	(0.267)	(157.362)	(1.803)
Year FE	Yes	Yes	Yes	Yes	Yes	Yes	Yes	Yes	Yes	Yes	Yes	Yes
Province FE	Yes	Yes	Yes	Yes	Yes	Yes	Yes	Yes	Yes	Yes	Yes	Yes
athrho	-0.108***		0.148***				-0.099***		0.172***			
	(0.025)		(0.030)				(0.026)		(0.035)			
N	10330		9768		2215		9632		4149		2099	
Log pseudo likelihood	-8587.1028		-6400.4488				-8028.5224		-4185.0971			
R^2^					0.115						0.113	

Note: Standard Deviation in parentheses.

***, ** and * signify statistical significance at 0.01, 0.5, and 0.1level respectively. athrho is the hyperbolic arctangent of the error correlation.

The cross effects for price in the whole sample and the subgroup of ever-smoked are not statistically significant and asymmetry in the direction or magnitude. In the subgroup of currently smoking, a price increase of SSBs will significantly reduce the amount of cigarettes smoked, indicating a complementary relation between SSBs and cigarettes in consumption and echoing the positive correlation between the amount of SSB intake and cigarette smoked in the subgroup of currently smoking observed from the result of sample selection model (Table 9). Generally, the cross effects of price are not statistically significant. So it is with the male sample. One possible explanation may be that the community-level price data in CHNS could not reflect the price information faced by a specific individual and lacks of variation across communities and over the survey period [22]. In sum, regarding the interdependence between SSB and cigarette consumption, conclusions drawn from the correlation of error terms for SUBP and SUR models are consistent with that drawn from Heckman sample selection models while the estimated cross effects of price provide only limited support for such interdependence.

About the own price effect, an increase in SSB price leads to a reduction in the possibility of consuming SSB frequently in the whole sample but has no significant effect on participation decisions in the subgroup of ever-smoked and currently smoking, consistent with the results of the sample selection models. As for cigarettes, the increase in cigarette price will encourage more smokers to quit smoking in the subgroup of ever-smoked but have limited effect in reducing the amount of cigarettes smoked daily for current smokers. The price effect differs significantly across different subgroups of cigarette consumers, in line with the findings of Gao et al. (2010). They examined the cigarette demand of Chinese with data from the CHNS and found that the price increase was more effective in discouraging participation in cigarette consumption in contrast with reducing the amount of cigarettes smoked. Such reduction varied remarkably across different demographic categories.

Notably, the demographic characteristics of SSB consumption are different from that of cigarette consumption. An increase in income encourages more participation and intake in SSB consumption in general, echoing the results of sample selection models, but induces an increase in the number of individuals who quit smoking. For current smokers, more income means more cigarettes smoked daily, supportive of the findings from Ren et al. (2020) that income could significantly increase the amount of cigarettes smoked by Chinese. Moreover, different from SSB consumption, cigarette consumption exhibits an inverted U-shaped correlation with age. A higher level of education and residing in urban areas correspond to less cigarette consumption while more SSB consumption.

## 6 Conclusion and discussion

This study finds that SSBs could be complements for cigarettes. In the whole sample, individuals who ever smoked once are more likely to be high-frequent SSB consumers with a higher level of average intake of SSBs per week. In the sample of those who are smoking currently, the more cigarettes individuals consume daily, the more likely for individuals to take in more SSBs. However, SSBs could also be substitutes for cigarettes. In the sample of those who ever smoked once, individuals who quit smoking are more likely to consume SSBs in comparison with those who are still smoking.

SSBs and cigarettes are correlated in consumption and such correlations vary across different groups, echoing the inconsistent findings regarding the interdependence between cigarettes and alcohol in consumption. As aforementioned, cigarettes and alcohol could be substitutes because they are both complements to leisure time [12] or complements in the case that consumers who go to bars would consume cigarettes and alcohol at the same time [13]. Research further pointed out that such opposite conclusions may derive from the group difference in cigarettes and alcohol consumption [12, 13]. In this study, we find that in the subgroup of ever-smoked individuals who quit smoking are more likely to consume SSBs, in line with the substitutable relation between cigarettes and alcohol. While in the subgroup of currently smoking, we observed a significantly positive correlation between the amount of SSBs and cigarettes consumed, corresponding to the complementary relation between cigarettes and alcohol. The findings of this study provide support for the interdependence between the consumption of different addictive substances and empirically validate the group difference of such correlation.

However, the reason behind the group difference remains unclear. One possible explanation, given the addictiveness of SSBs and cigarettes, may result from the fact that individuals’ SSB or cigarette consumption is at various stages in the process of dependence formation. WHO (2021) pointed out that there is no sugar or tobacco dependence at the initial stage of SSB or cigarette consumption. At this point of time, individuals could easily give up smoking and turn to SSBs or vice versa and SSBs and cigarettes are substitutes correspondingly. Similarly, Decker and Schwartz (2000) finds that smokers who quit smoking in face of a price increase of beer are mostly “social smokers” or “occasional smokers”. However, as time goes by, individuals who periodically consume SSBs or cigarettes have to gradually increase the amount of SSB intake or cigarettes smoked to maintain the same level of utility due to tolerance. Once individuals develop sugar or tobacco dependence, they compulsively consume a certain amount of SSBs or cigarettes every day, even if they realize the health risks related to addictive consumption. At this time, SSBs and cigarettes are complements.

The identification of such correlations emphasizes the importance of applying a systematic approach to explore the demand for the two goods due to the statistical efficiency. Such correlation would also affect the estimation of the own price elasticities for SSBs or cigarettes. In light of the diversity of addictive substances, further exploration of the interdependence between SSBs and other addictive substances should be carried out in the future. For example, besides cigarette addiction, China faces a continuous increase in alcohol consumption which has led to a gradual decline in the lifelong abstinence rate and a rapid increase in the proportion of alcoholic liver disease [55]. Conducting systematic research on the consumption of alcohol, cigarettes, and SSBs is of great significance.

The first policy implication of our study is that the complementary relation between SSBs and cigarettes for current smokers aggravates individuals’ health risks and thus necessitates more attention from policymakers. What’s more, policies aiming at reducing cigarette consumption may have a double dividend effect on health given the simultaneous decrease in SSB consumption for the current smokers. Second, although there is no difference between former and current smokers in the possibility of being a high-frequent SSB consumer, given that SSBs are addictive substances and periodical consumption may lead to sugar dependence, former smokers’ substituting SSBs for cigarettes may also pose a threat to individuals’ health. Such substitution may offset the health effect of policies targeting at reducing cigarette consumption and vice versa.

In sum, the correlation of SSBs and cigarettes in consumption underlines the interdependence of public policies for the two goods. Our findings help policymakers to better coordinate diet interventions targeting at curbing SSB consumption with policies aiming at reducing cigarette demand. Especially in the context of China, no diet interventions concerning SSB consumption have been implemented yet except for nutrition education. Systematic interventions in the consumption of addictive substances should be considered. Lastly, it should be noted that caution should be exercised when generalizing our findings to other countries given the inherent heterogeneity across cultures and nations.
